# Multiscale modelization in a small virus: Mechanism of proton channeling and its role in triggering capsid disassembly

**DOI:** 10.1371/journal.pcbi.1006082

**Published:** 2018-04-16

**Authors:** Juan Francisco Viso, Patricia Belelli, Matías Machado, Humberto González, Sergio Pantano, María Julia Amundarain, Fernando Zamarreño, Maria Marta Branda, Diego M. A. Guérin, Marcelo D. Costabel

**Affiliations:** 1 Departamento de Física (DF), Universidad Nacional del Sur (UNS), Bahía Blanca, Argentina; 2 DF-UNS, Grupo de Biofísica, Instituto de Física del Sur (IFISUR, UNS/CONICET), Bahía Blanca, Argentina; 3 DF-UNS, Grupo de Materiales y Sistemas Catalíticos (GRUMASICA), IFISUR, Bahía Blanca, Argentina; 4 Grupo de Simulaciones Biomoleculares, Institut Pasteur de Montevideo, Montevideo, Uruguay; 5 Instituto Biofisika (UPV/EHU, CSIC), Department of Biochemistry and Molecular Biology, University of the Basque Country (EHU), Barrio Sarriena S/N, Leioa, Vizcaya, Spain; Max Planck Institute for Biophysical Chemistry, GERMANY

## Abstract

In this work, we assess a previously advanced hypothesis that predicts the existence of ion channels in the capsid of small and non-enveloped icosahedral viruses. With this purpose we examine *Triatoma Virus* (TrV) as a case study. This virus has a stable capsid under highly acidic conditions but disassembles and releases the genome in alkaline environments. Our calculations range from a subtle sub-atomic proton interchange to the dismantling of a large-scale system representing several million of atoms. Our results provide structure-based explanations for the three roles played by the capsid to enable genome release. First, we observe, for the first time, the formation of a hydrophobic gate in the cavity along the five-fold axis of the wild-type virus capsid, which can be disrupted by an ion located in the pore. Second, the channel enables protons to permeate the capsid through a unidirectional Grotthuss-like mechanism, which is the most likely process through which the capsid senses pH. Finally, assuming that the proton leak promotes a charge imbalance in the interior of the capsid, we model an internal pressure that forces shell cracking using coarse-grained simulations. Although qualitatively, this last step could represent the mechanism of capsid opening that allows RNA release. All of our calculations are in agreement with current experimental data obtained using TrV and describe a cascade of events that could explain the destabilization and disassembly of similar icosahedral viruses.

## Author summary

Plant and animal small non-enveloped viruses are composed of a capsid shell that encloses the genome. One of the multiple functions played by the capsid is to protect the genome against host defenses and to withstand environmental aggressions, such as dehydration. This highly specialized *capsule* selectively recognizes and binds to the target tissue infected by the virus. In the viral cycle, the ultimate function of the capsid is to release the genome. Observations of many viruses demonstrate that the pH of the medium can trigger genome release. Nevertheless, the mechanism underlying this process at the atomic level is poorly understood. In this work, we computationally modeled the mechanism by which the capsid senses environmental pH and the destabilization process that permits genome release. Our calculations predict that a cavity that traverses the capsid functions as a *hydrophobic gate*, a feature already observed in membrane ion channels. Moreover, our results predict that this cavity behaves as a *proton diode* because the proton transit can only occur from the capsid interior to the exterior. In turn, our calculations describe a cascade of events that could explain the destabilization and dismantling of an insect virus, but this description could also apply to many vertebrate viruses.

## Introduction

Many small viruses are minimalistic structures composed of a spherical proteinaceous capsid constituted by several replicas of one or few proteins that enclosing the viral genome. The capsid plays many different roles, such as recognizing its own genome and provoking its encapsidation, protecting and transporting the genome in the extracellular space, and specifically recognizing and invading cells of the target tissue.

The viral life cycle culminates in a mature viral structure that is able to infect new cells. During such processes, capsid destabilization is a key structural change that is required to release the genetic material into the cytoplasm of the target cell. This destabilization can be triggered by interactions with cell receptors or promoted by chemical or physical conditions, such as pH and temperature changes.

The *Picornavirales* order includes many vertebrates, invertebrates, and plant viral families of small non-enveloped viruses containing a +ssRNA genome [1]. The viruses’ common structural characteristics are an icosahedral oligomeric viral capsid made of 60xT repeats (T = 1 or T = 3) and a capsid size of approximately 30 nm in diameter. The structural proteins are named VP1-4, with VP1-3 exposed to the exterior particle surface and VP4 in the interior in contact with the genome. The pentameric sub-unit composed of 5xVP1-4, called the *penton* or *pentamer*, constitutes a capsid disassembly and assembly intermediate. Many of the members of this viral order have been studied extensively, either because they are agent diseases in humans, such as poliomyelitis, hepatitis A, and common cold, or in livestock and crops. One member of the picornavirales family that nucleates insect viruses and has economic and epidemiological importance is *Dicistroviridae* [2]. Within this family is *Triatoma virus* (TrV), the type species of the genus *Triatovirus*, which is the focus of this work. Another member is *Cricket paralysis virus* (CrPV; type species of the *Cripavirus* genus), the atomic structure of which demonstrated organizational similarities between these viruses and picornaviruses hosted by vertebrates [3,4].

In this work we model the process by which a viral capsid could sense the environmental pH and the mechanism that could promote the destabilization and opening of the protein shell. The experimental data that guide our calculations come from previous structural, stability and disassemble studies done on TrV [5–7].

These data showed that this viral capsid is stable at acidic pHs [6] while disassembles under alkaline conditions, and that the genome is the molecule that drives its own release by cracking the protein cage [7]. Due to the genome is isolated at the capsid interior, these data suggests that some kind of interchange, either of solvent or solutes, may occur through the protein shell that mine the equilibrium reached during the process of virus assembly.

In this report, we explore the hypothesis of the existence of ion channels along the five-fold symmetry axes in the capsid of small icosahedral viruses [8,9]. We tested this hypothesis concerning the structure of the TrV capsid (Protein Data Bank code, 3NAP) using techniques, such as molecular dynamic (MD) simulations for channel hydration, quantum mechanic (QM) simulations to study proton transport and coarse-grained (CG) simulation of the full TrV capsid to assess structural stability. Overall, this report presents a simulation study that was conducted on a macromolecular system encompassing more than six orders of magnitude in time and size, ranging from subatomic distances to a system representing nearly 6 million atoms.

Our study provides a novel and simple model of the concerted processes leading to TrV genome release, i.e., pH sensing by means of a proton channel spanning the viral capsid along the five-fold axes. Draining of protons from the capsid interior disrupts the electrostatic equilibrium, destabilizing the structure and eventually enabling the exit of the genome.

## Results

### Hydrophobic gate in the five-fold cavity

**Fig 1:**
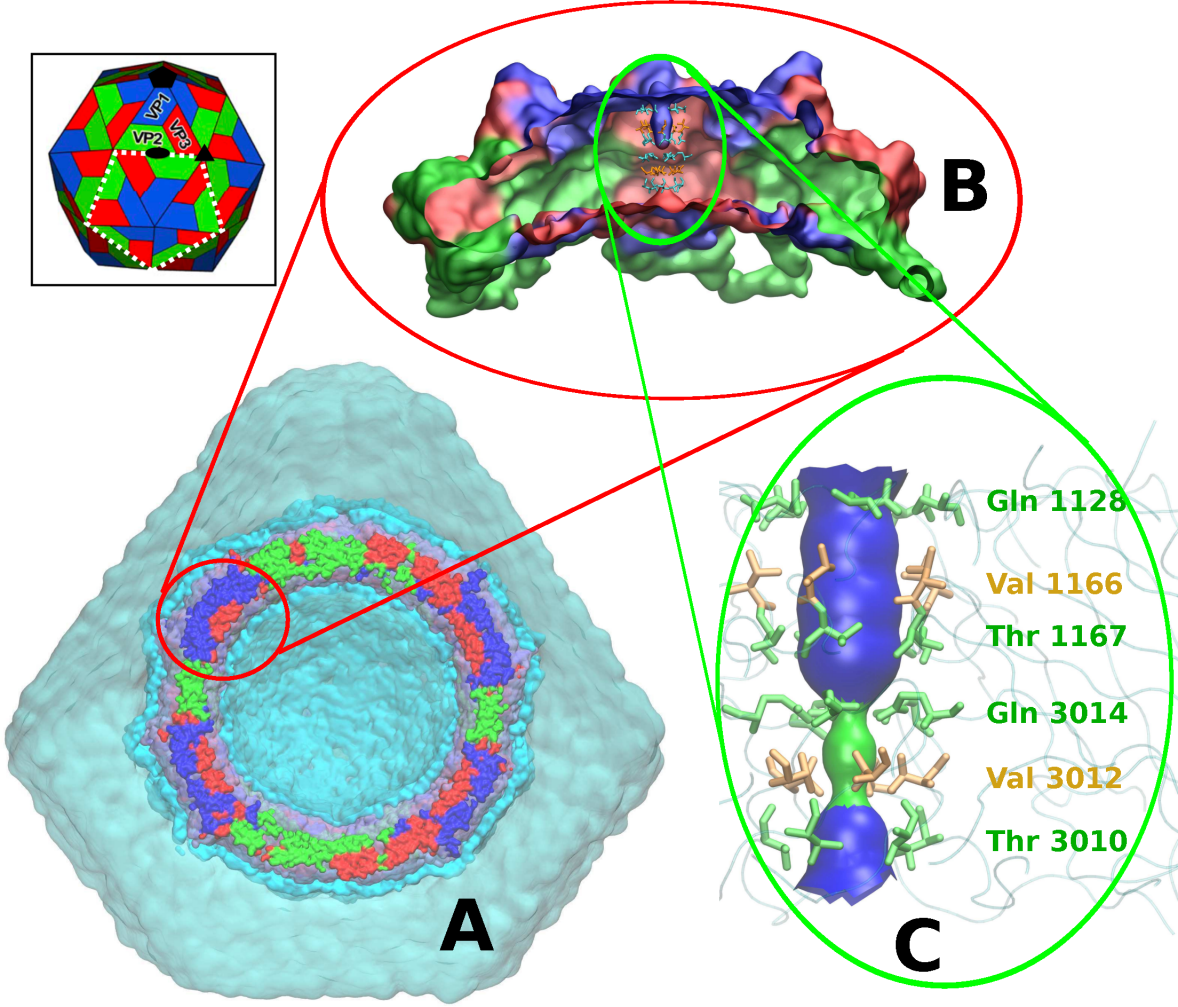
Protein and solvent models of the MD simulations. **A.** The whole system contained in an icosahedral box. Clipped TrV capsid representation with viral proteins colored in blue for VP1, green for VP2 and red for VP3. Water molecules are represented as a continuous surface, ice blue for atomistic SPC waters, cyan for coarse-grain WT4 beads and transparent cyan for coarse-grain WLS (see S3 Fig). **B.** Side view of the clipped TrV capsid penton (5xVP1-3) in a surface representation (proteins are colored with the same code as in A). **C.** Simplified representation of the pore found along the five-fold symmetry axis of the TrV capsid. Amino acids lining the cavity are represented as sticks (green are polar amino acids and yellow are non-polar). The main chain of the polypeptide is sketched in light blue. The internal narrowest region of the cavity is shown as a green surface, and the wider pore regions with a diameter up to ca. 0.9 nm are colored blue. Inset: The TrV capsid is composed of 12 pentamers, each composed of 5xVP1-3 proteins. The white dashed line indicates the frontier of one pentamer (or *penton*).

**Fig 2:**
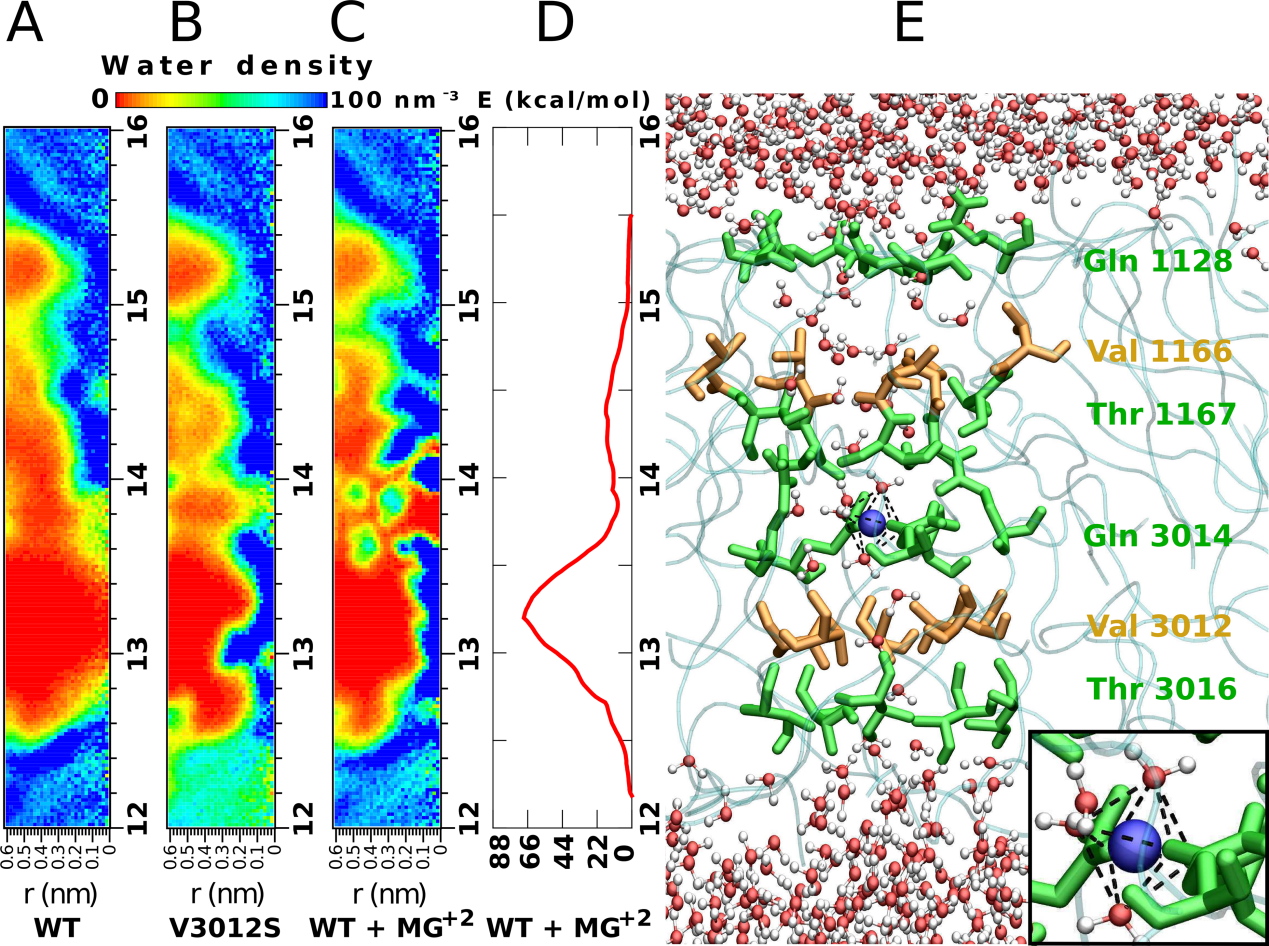
MD modeling of the hydrophobic gate, structural ion and pore hydration. **A.** Water density map corresponding to the simulation of the five-fold cavity of the TrV WT structure. Regions colored in deep red are completely devoid of water molecules during the entire 100-ns simulation. The zone between the capsid radial distances of 13.0 and 13.4 nm constitutes the *hydrophobic gate*. The horizontal scale in panels A-C corresponds to the radial distance measured perpendicular to the five-fold symmetry axis. **B.** Water density map of the simulation of the mutated TrV Val3012Ser. **C.** Water density map of the simulation of TrV with a magnesium ion (Mg^2+^) inside the pore. **D.** Potential of the mean forces (PMF) of the Mg^2+^ alongside the pore. The higher maximum coincides with the position of the Val 3012 ring, while the second maximum matches with the position of Val 1166. The free energy minimum corresponds to the position of residues Gln 3014. **E.** Lateral view of the pore showing the bulk solvent water molecules (top and bottom parts) and water molecules that enter the narrow cavity region when a divalent ion (blue sphere) is present. Amino acids lining the pore are colored according to their properties, polar are green and non-polar orange. (INSET) Mg^2+^ octahedrically coordinated with three oxygens of water molecules and three symmetry-related Oε1 from Gln 3014 side chains (mean distance Mg^2+^-O = 0.21 nm ±0.01 nm).

Using the crystal structure of TrV as an initial model [10], an MD simulation of the solvated capsid in an atomistic representation was performed with the atomic and CG model for solvent molecules in different hydration regions (Fig 1A). A 50-ns calculation showed an inhomogeneous hydration pattern across the five-fold axis of the capsid pore. Hence, to further characterize the solvent inside the pore over a longer time scale, a simulation of a single pentamer around the five-fold symmetry axis (Fig 1B) was carried out at an atomistic level, as described in the Materials and Methods section. This 100-ns calculation showed that the cavity had three regions with a solvation pattern that differed from the bulk solvent. These regions spanned the five-fold cavity along the axis by six annuluses of symmetry-related amino acids (Fig 1C). The middle region located at the narrowest portion of the five-fold cavity remained devoid of water molecules (green zone in Fig 1C). This effect was observed within the cavity lumen surrounded by the two rings formed by Gln 3014 and Val 3012. We will refer to this region as the *hydrophobic gate*. Analysis of the pore radius indicated that the hydrophobic gate coincides with the maximum constriction point of the cavity, featuring a radius of nearly 0.3 nm. From this point towards the capsid exterior and interior, the cavity widened but did not exceed a diameter of 0.9 nm (blue regions in Fig 1C). Along the entire simulation of 100 ns, the hydrophobic gate of ca. 0.4 nm in length remained dehydrated (Fig 2C). A similar calculation, but with a Val3012Ser mutation, confirmed that solvent depletion was caused by the hydrophobic character of the valine annulus. Indeed, this simulation with a serine at position 3012 resulted in a more hydrated pore, even though the pore radius had the same diameter as in the native structure (Fig 2B).

The electron density map of TrV, computed with experimental X-ray diffraction data, showed a spherical bulge on the five-fold symmetry axis at the level of Gln 3014. This electronic density was attributed to the presence of a putative metal ion [10]. Based on these data, we considered whether a magnesium ion, Mg^2+^, inside the pore would modify the hydration state within the cavity. To determine energetically favorable ion positions along the five-fold axis, we performed a MD simulation with the umbrella sampling method (USM) [11]. The main result was the extraction of the potential of mean force (PMF), which provided the ΔG for the binding/unbinding process. Fig 2D shows that the most favorable position for this ion is near the Gln 3014 ring, since this point corresponds to a free energy minimum (Fig 2D). At this position, the Mg^2+^ is coordinated to three Gln 3014 side chain carbonyl oxygen atoms (Oε1) plus three water molecules with octahedral symmetry (Fig 2E inset). Under this condition, several water molecules filled the cavity, thereby chaining the external solvent to the solvent at the inner region (Fig 2C and 2E). We will refer to this water structure as *water wire*.

Concerning the umbrella sampling simulations, a close inspection of each window showed that the ion was fully hydrated only at the minimum of the energy profile. Under this situation, the hydration of the cavity was complete.

Regarding these calculations, ions initially located close to both cavity ends were expelled out within a few nanoseconds towards the bulk solvent. As expected, ions with an initial position close to the ring formed by Gln 3014 remained stable throughout the whole 100 ns of the simulation. The local energy minimum corresponding to this position was approximately 11.3 kcal/mol (Fig 2D).

Simulations in which Val 3012 was mutated by serine and in which Mg^2+^ was positioned inside the pore in the minimum observed free energy profile showed immediate hydration of the pore (in approximately 5 ns). However, in the simulation of the wild-type pentamer, the pore was dehydrated during the entire 100 ns of simulation. Thus, the water molecules had sufficient time to enter the hydrophobic region during the length of the wild-type simulation.

We chose Mg^2+^ to model the unassigned electron density within the five-fold cavity of TrV because this ion is the most abundant among biological divalent cations. However, an equivalent MD simulation performed by replacing Mg^2+^ with Ca^2+^ provided similar results.

### pH sensor through a Grotthuss-like mechanism of proton channeling

**Fig 3:**
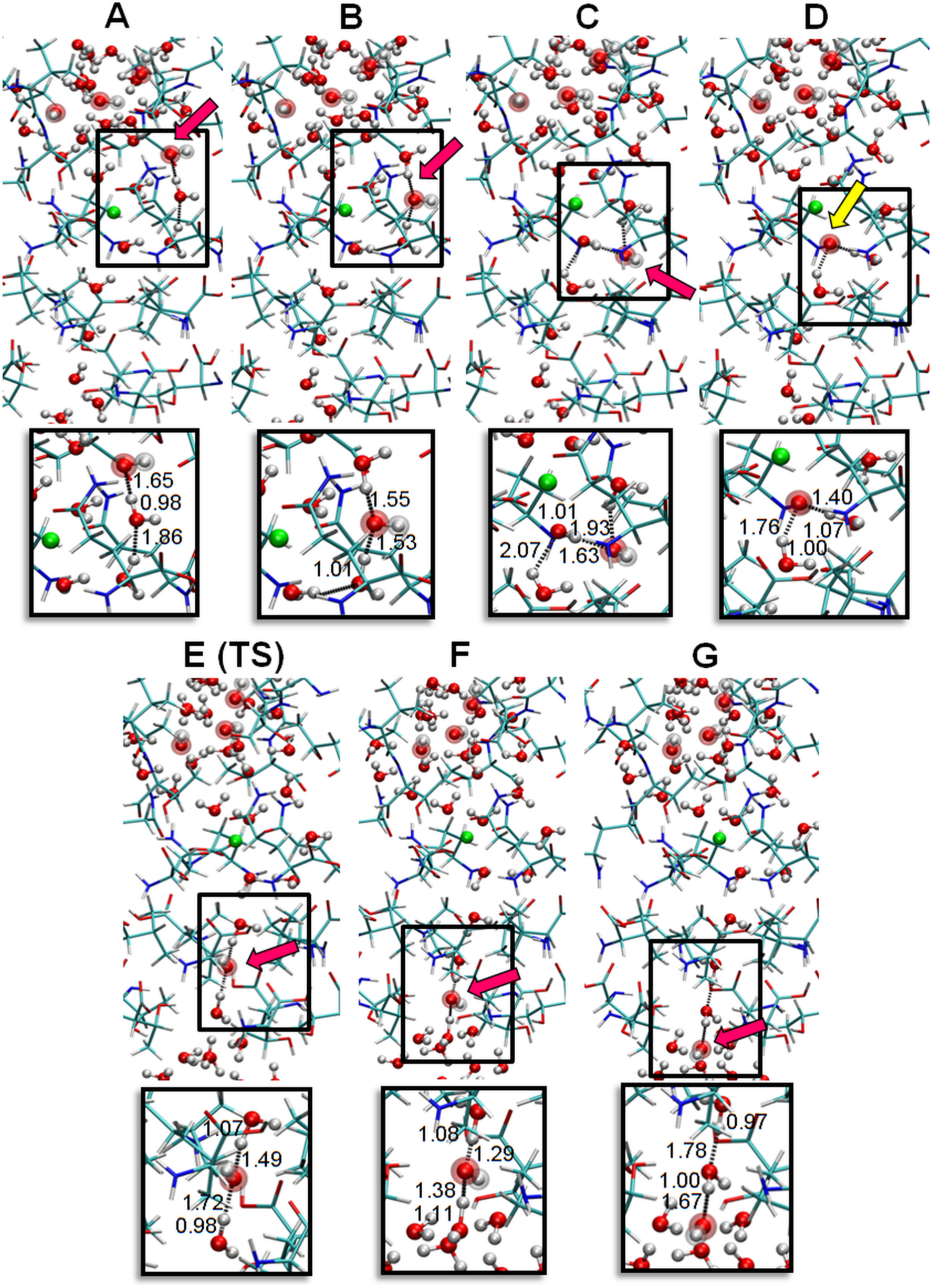
Proton migration through a Grotthuss-like mechanism. The hydration promoted by the ion located in the five-fold cavity allows proton migration through the cavity. Protein atoms are indicated by a stick model with red and blue segments. Oxygens from water (H_2_O) are denoted by small red spheres, and oxygen from hydroxyl ions (OH^-^) are large red spheres. Solvent protons are small white spheres, and protein protons are white sticks. The Mg^2+^ is highlighted as a green sphere. Panels A-G show the sequence of events used for the QM calculations for proton transport induced by a pH gradient. Four OH^-^ located in the outer solvent region represent a high pH relative to the capsid inner solvent region. Red arrows point to the position of OH^-^ at each step of the simulation, and these points correspond to the H^+^ hole jumping stepwise from the top to the bottom. The yellow arrow indicates the water coordinated to the metal ion that participates in the inner part of the water wire. Panel E corresponds to the transition state (TS) between configurations D and F, in which the H^+^ hole overcomes a low energy barrier towards the capsid interior. Lower panels show the distances (in Å) at each step between the atoms participating in the proton transfer. For clarity, only a subset of the 536 atoms involved in the calculation was included in these panels.

Starting with the water structure determined by the MD simulations and to simulate a basic pH, one proton (H^+^) from each of the four water molecules located in the outer zone of the channel were removed (Fig 3A), resulting in a ratio of OH^-^/H_2_O = 4/38. Geometry optimization was then performed (see Quantum multilayered simulation in Materials and Methods). The sequence shown in Fig 3 corresponds to different steps obtained during the QM simulation. In this process, one of the OH^-^ captures a H^+^ of the closest water molecule, which in turn is bonded to another innermost water molecule (Fig 3A–3D). The concerted proton transfer continues until the OH^-^ is bound to the Mg^2+^ cation (Fig 3D). Up to this point, protons transport (or the corresponding proton hole movement in the opposite direction) occurs downhill in energy. However, the proton hole transferred from the OH^-^ bonded to Mg^2+^ through two waters located towards the capsid interior is not energetically favored (Fig 3E TS), probably because the Mg^2+^ cation has a higher affinity for OH^-^ species than for H_2_O molecules. Nevertheless, the calculated activation energy barrier is low, at 1.7 kcal, approximating the kinetic thermal energy of an atom or molecule at room temperature (3/2 K_B_T~0.9 kcal). The final configuration after this transition state is shown in Fig 3E. The highest energy requirement was clearly associated with the deprotonation of the nonconsecutive water molecule, that is, the formation of OH^-^ at the second water molecule below the Mg^2+^. From this geometric configuration, the concerted proton hole transfer occurred downhill up to generate an OH^-^ at the end of the water wire immediately at the frontier of the bulk solvent (Fig 3G).

Two similar simulations, a calculation with the outer three OH^-^ in different positions and the other including only a single OH^-^ located at the outer molecule of the water wire, gave the same concerted proton transfer until the OH^-^ bound to the Mg^2+^ cation (see S1 Fig).

At this point, we must highlight that during the geometric optimization, the three remaining hydroxyls placed at the outer zone of the capsid channel maintained their identities, indicating that none of the OH^-^ captured any protons from vicinal water molecules. Most likely this happened because hydroxyls interact with waters in their hydration sphere through strong hydrogen bonds and breaking one water bond would require more than 100 kcal/mol [12]. Other authors have previously reported this kind of interaction [13–16]. The same solvation was observed at the end of the proton hole transfer when the hydroxyl was generated in the interior of the channel.

**Fig 4:**
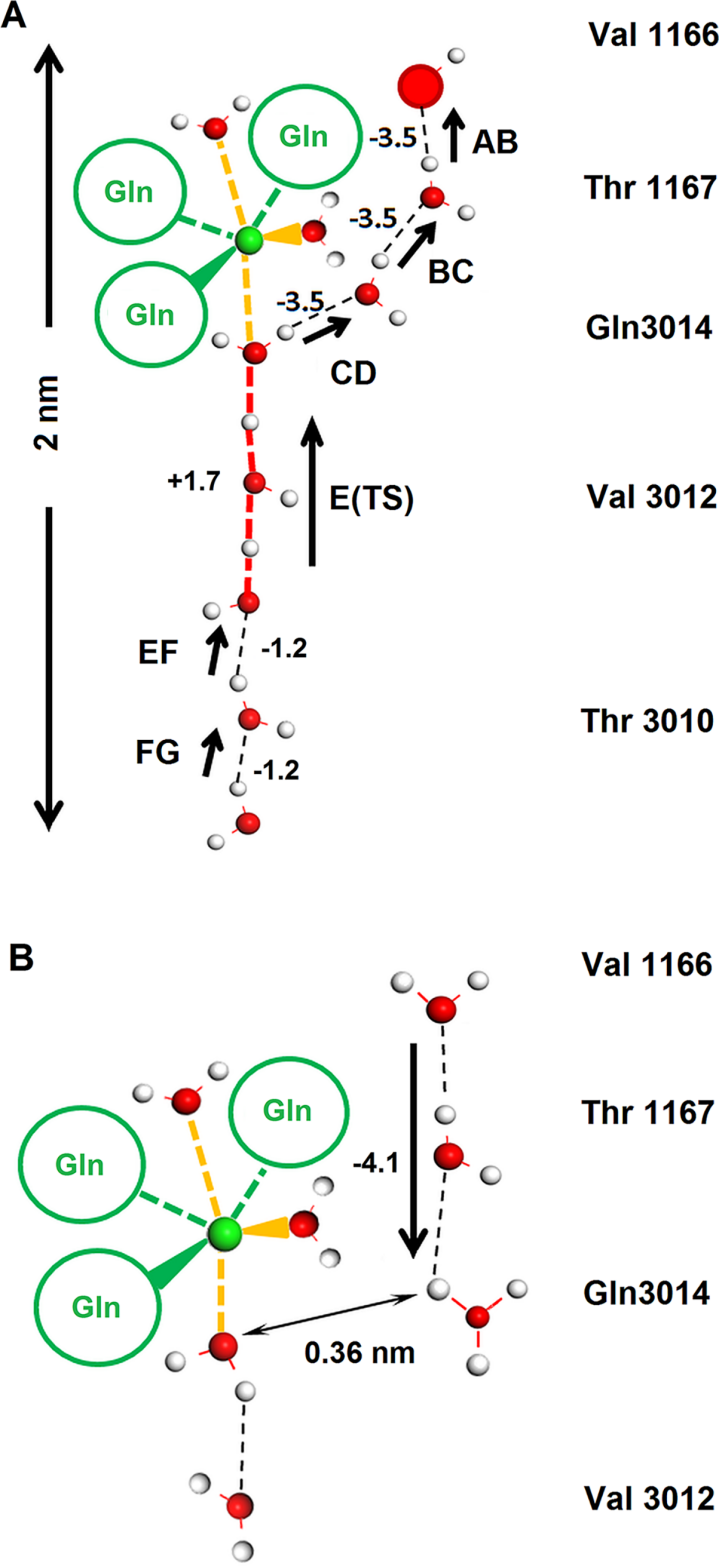
Schematic representations of the Grotthuss and Grotthuss-like mechanism for proton channeling. The amino acid labels indicated on the right side serve as positional references. The Mg^2+^ (green sphere) is coordinated with three water molecules and three Oε1 side chain oxygen atoms based on the symmetry-related Gln 3014 (green circles). **A.** When the external capsid pH is basic with to respect the internal region, protons jump between adjacent waters as indicated by the arrows. Under this condition, H^+^ holes can migrate from the capsid exterior (overcoming a low energetic barrier), showing a concerted Grotthuss-like mechanism of proton jumps. This sequence corresponds to panels A-G of Fig 3. **B.** When the outer solvent region is acidic with respect to the capsid interior (higher hydronium concentration), proton migration occurs between hydronium and H_2_O molecules. A proton that binds to a hydronium in the upper bulk solvent can go down two steps along the external portion of the water wire (ΔE ~ -4.2 kcal/mol). When this proton migration occurs, it stops at the water close to the water molecule coordinated to the Mg^2+^. From this point down, the water wire is disrupted due to the increase in distance between the hydronium and the inner-most proximal water molecule (~0.36 nm), which is precisely the one linked to the metal cation. Breaking of the water wire constitutes the electrical impediment for the proton to proceed downward and reach the capsid internal solvent. Positive and negative numbers indicate the approximate energy value (in kcal) that corresponds to each proton jump. For clarity, only a subset of the 536 atoms involved in the calculation was included in these panels.

The migration of H^+^ from an inner water molecule to another located toward the capsid exterior agrees well with a Grotthuss-like mechanism called "*Proton Holes*" [17]. Fig 4A displays this migration in a sequence of seven steps that covers a pathway of approximately 2 nm along the water wire. In the initial portion of the trajectory, the proton hole executes three energy favored jumps (ca. -3.5 kcal each). It reaches the inner most water molecule that is coordinated to the Mg^2+^ ion. From this point forward, the proton hole must overcome a low barrier (of ca. +1.7 kcal) that covers two units of the water wire. Finally, the proton reaches the water at the internal bulk solvent frontier through two favored jumps (ca. -1.2 kcal each).

The three situations we analyzed here have in common the position of the outer hydroxyl from which starts the proton hole transfer. Then, these situations illustrate one way how a concerted proton hole transport can occur. If the position of the initial hole-donor would be different, most likely the proton hole transfer through the water wire pathway will looks different, certainly displaying slightly different energy proton-jumps.

A similar simulation was performed but with neutralization of the negative hydroxyl charges by placing four Li^+^ ions in the outer water bulk zone. This new simulation provided the same result obtained without lithium ions, indicating that the proton migration was due to the pH gradient and not to a charge gradient.

Another simulation was performed by replacing Mg^2+^ with Ca^2+^. Additionally, the expected increase in the oxygen cation distances was due to the larger size of calcium (0.24 nm compared with 0.21 nm for Mg^2+^), and both the structure and the same concerted mechanism occurred in a completely analogous fashion.

To study the influence of external acidic pH, a similar model to that employed in the former situation was designed by replacing the external OH^-^ with H_3_O^+^. In this condition, the calculation started with the hydronium ions in equivalent positions to the OH^-^ ions shown in Fig 3A. In the first simulation with a basic external pH, a transfer of the H^+^ hole occurred; in the reverse situation with an acidic external pH, the inverse process of classical Grotthuss happened, that is, the proton from the hydronium jumped twice to the closest internal water molecules (ΔE ~ -4.1 kcal/mol). Nevertheless, proton internalization did not occur because proton migration stopped immediately before reaching the water molecule coordinated to the Mg^2+^ cation (Fig 4B). The water wire near the metal ion becomes disrupted due to an increase in the distance between the hydronium and the inner water molecule. This distance is approximately 0.36 nm (Fig 4B). This disruption was most likely due to an electrostatic repulsion between the cation and the hydronium.

### Imbalance of the internal charge promotes capsid dismantling

Upon encapsidation, the viral RNA is highly condensed due to the neutralization action of counterions such as Ca^2+^, Mg^2+^, K^+^ and Na^+^. Although under physiological conditions (i.e., ~0.15 M of simple electrolytic ions) other negative ions are also present, like Cl^-^ or OH^-^, within the capsid of a mature virion the charge balance must be close to neutrality. This equilibrium situation would be maintained after completion of the process of RNA encapsidation and while the virus remains in the intracellular space (with a neutral or lightly acidic pH). When the virus abandons the infected cell, the pH of its environment changes, and upon binding to the receptor of the target cell, the capsid may allow ion permeation. If this occurs, the equilibrium between positive counterions and RNA will be altered. Our QM simulation indicates that a high pH at the capsid exterior and Mg^2+^ inside the cavity would drive a Grotthuss-like effect of proton exit, producing an internal charge imbalance and structural instability. This charge excess can impact to one or more of the multiple molecules located at the capsid interior: OH^-^s, Cl^-^ and other anions in solution, and deprotonable amino acids and/or RNA bases located at fixed positions. For instance, the amino termini of VP1-3 proteins are part of the internal capsid wall in direct contact with the RNA [10], and these termini could be deprotonated when the pH is of about 8.2 [18]. These 180 titratable groups are located around the three- and five-fold axes, and distributed in a thin spherical shell (see S2 Fig and S1 Table). If we also count the 60 copies of the N-teminus of VP4 peptide that are disordered and in contact with the genome [5], all make a total of 240 groups that can be deprotonated when the internal pH gets alkaline. *Such distribution of chargeable groups embedded into an almost solid matrix could produce a big increase in internal pressure even when the repulsion would happen among few groups situated in a small region*. Unfortunately, experimental evidence suggesting which molecular species are predominantly affected is not currently available. Thus, we attempted to phenomenologically model this situation at the CG level. For this aim we first ran a CG simulation of an empty capsid using the method presented in ref. [19]. Then, we generated an electrostatic internal repulsive force by placing Cl^-^ ions, simulating internal charges inside the capsid.

**Fig 5:**
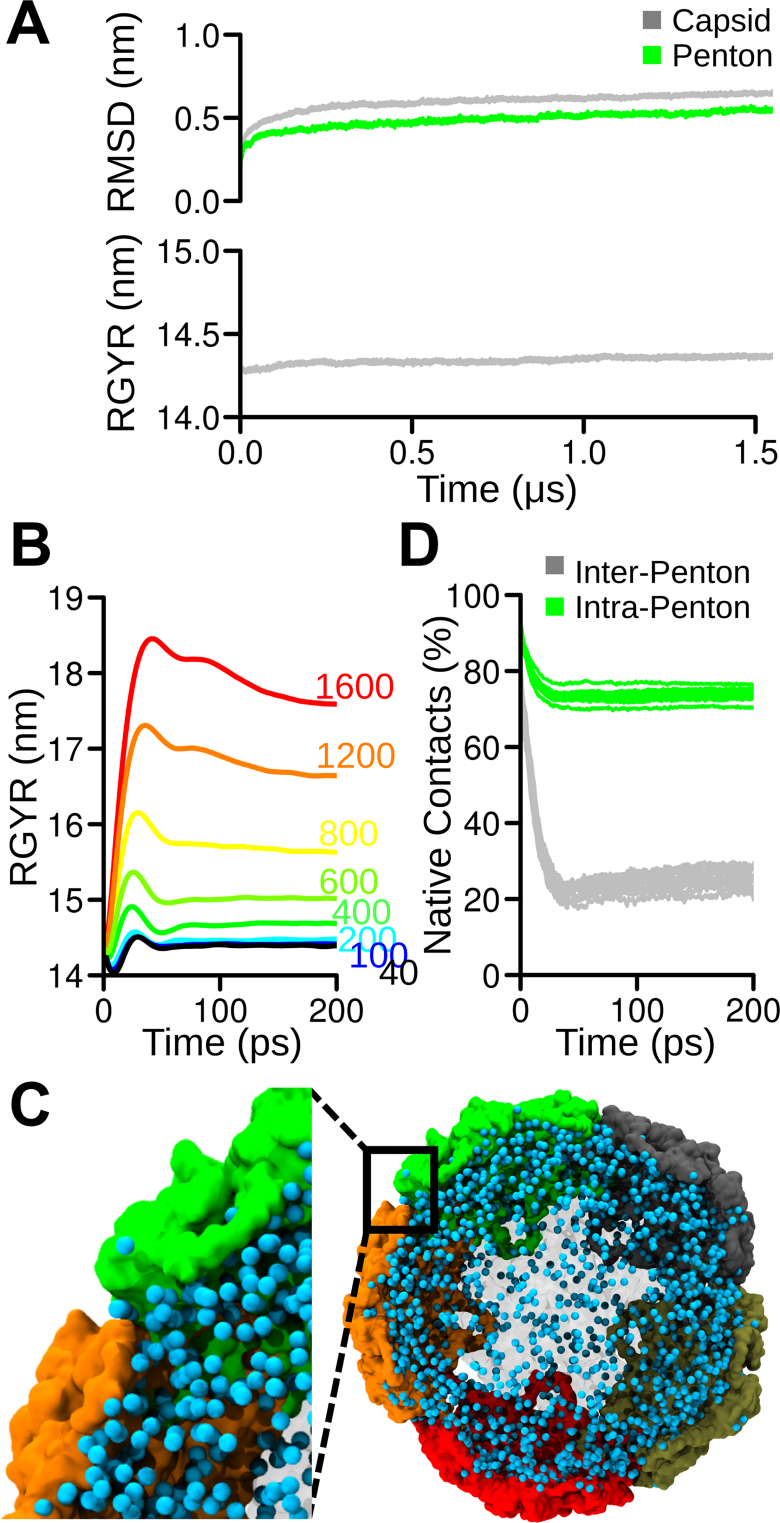
CG modeling of the TrV capsid and electrostatic destabilization. **A.** RMSD (top) and the gyration radius (bottom) calculated for the amino acid alpha carbon positions along the MD trajectory. The RMSD of the entire capsid and one single penton are presented in gray and green, respectively. **B.** Gyration radii of the capsid loaded with increasing amounts of Cl^-^ in the interior. The number of anions is indicated in each curve by the same color. **C.** Molecular representation of the destabilized capsid. Solvent and pentons in the front and back of the plane are semitransparent for improved visualization. The remaining pentons are depicted in different colors. The inset illustrates the preferential separation between pentons that allows the exit of Cl^-^. **D.** Protein-protein contacts monitored along the simulation containing under extreme conditions 1600 Cl^-^ within the capsid core (corresponding to an internal charge density of 2.3x10^-4^ e^-^/Å^3^). Inter-penton and intra-penton contacts are presented in gray and green, respectively.

We performed a 1.5-μs long simulation, along which the global structure of the capsid experienced a relatively high change in RMSD of the alpha carbon positions (Fig 5A), which was mainly related to a loss of the initial symmetry. This effect was reflected mostly at the global level and, to a lesser extent, on individual pentons. Indeed, calculation of the gyration radius along the simulation revealed deviations from the experimental value of only ~0.1 nm (Fig 5B).

The stabilized CG structure after 1.5 μs of simulation was used as a starting point for a series of simulations with progressive substitution of supra coarse-grained water molecules (WLS; see S3 Fig), with Cl^-^ ions at the capsid interior being located at random positions. This set up attempts to mimics the conditions after alkalinization has happened. It is worth noticing that this process is intended only to roughly mimic the progressive increment in negative charges at the core. Since the intrinsic entropy loss in the coarse-graining process makes impossible a one-to-one energetic comparison, the number of ions used must not be considered as an actual number of charge carriers but rather as a mean to progressive increase the internal electrostatic repulsion. This charge imbalance produces repulsions among ions, generating an internal pressure. The impact of this pressure was evaluated through an MD simulation in terms of the capsid radius of the gyration (RG) increase. Incorporating less than 200 anions did not result in a significant change in the RG. Only a small oscillation was observed, which was likely ascribable to the re-stabilization of the system after the replacement of water with ions in the interior. After this oscillation, the radius returned to its initial value (Fig 5B). However, further increasing the number of ions resulted in a permanent increase in the RG of the particle, which was likely related to the number of ions impinging on the inner surface. Arbitrarily increasing the number of ions over 200 resulted in large distortions associated with a rapid increase in the RG. At these high values of non-neutralized internal charge, the internal pressure opened crevices that enabled the passage of ions (Fig 5C). Notably, comparison of the conservation of native protein-protein contact inter and intra pentons indicated that the capsid cracked preferentially at the interface between pentons, while the pentons showed a preference for maintaining their structure, which was in agreement with mass spectrometry and AFM nanoindentation experimental data previously reported by our group [7]. It is worth to notice that the CG simulations were performed in absence of Mg^2+^ (see Methods). However, this showed (*a posteriori*) not relevant artifacts, as the destabilization of the capsid occurs along the inter- and not intra-pentamer protein-protein interfaces, in agreement with our previous experimental results [7]. This is also in agreement with the absence of electronic density along the five-fold axis found in the structure of the empty capsid [20].

## Materials and methods

### MD simulations

The atomic 3-dimensional structure of TrV capsid determined through X-ray crystallographic techniques at a 2.5 Å resolution (PDB id 3NAP), excluding all solvent molecules, was used as starting point [10].

All simulations were performed with the program GROMACS 4.6.5 [21] using a multiscale approach. We used the Gromos53a6 [22] Force-Field and Single Point Charge (SPC) [23] water model for atomistic detail and the Sirah Force-Field for coarse-grain detail [24]. This multiscale approach uses three levels of representation of the solvent (see S3 Fig). To construct the system, we used the X-ray structure of the capsid solvated with 1.2 nm of SPC water shell. After the first atomistic solvation, a thick shell of 2.5 nm CG solvent [25] was added at the exterior and interior of the capsid. In both solvent regions, water molecules were randomly substituted with Na^+^ and Cl^-^ ions to neutralize the total charge and to mimic an ionic strength of 150 mM. The space remaining in the virus interior and between the capsid exterior and the box was solvated using a WLS model (see S3 Fig). This simulation was compared with a second set of simulations containing only a single penton (i.e., including one single five-fold symmetry axis) solvated with a 1.2-nm-thick shell of SPC water and the rest of the dodecahedral box/cell solvated using CG solvent. Periodic boundary conditions were applied using a dodecahedral with a sufficient size to maintain a distance of 1.5 nm between the proteins and the cell walls.

The multiscale approach used consisted of simulating the protein pentamer and a thin layer of water (1 nm thick) around it at an atomistic level, while the rest of the water molecules were simulated using the WT4 water model of the Sirah Force-Field. The simulations were performed in a physiological solution of NaCl at a concentration of 0.15 mol/liter and MgCl_2_ at a concentration of 0.015 mol/liter. In the case of the full capsid, a third layer was added beyond the WT4 layer. For this third level with less detail, WLS water molecules from the Sirah Force-Field (www.sirahff.com) were used.

To analyze capsid dismantling, simulations were performed by transforming the X-ray coordinates of the capsid to CG using SIRAH tools [26]. The CG protein was solvated with a 2.5-nm water shell, the ionic strength was set to 150 mM and the empty space was filled with CG water as in the triple layer case described above. The space remaining in the capsid interior and between the exterior and the walls of the computational box were solvated with supra coarse-grained water molecules (WLS; see S3 Fig). To induce capsid destabilization by electrostatic forces, we performed a series of CG simulations, in which we progressively substituted CG solvent molecules on the interior side with Cl^-^ ions and with Na^+^ on the exterior side of the capsid to maintain the electroneutrality of the simulation box. Within the coarseness of our simulation approach, Mg^2+^ ions at the five-fold axis were not considered because of two reasons: i) there was no available CG model for ions coordinated to both water molecules and oxygen atoms from amino acids, and ii) the CG model for ions were larger than the atomistic models because they included the first solvation shell and there was no space to harbor a structure such as a Mg^2+^ coordinated to six molecules within the hydrophobic gate.

A Verlet scheme was used to generate the neighbor search list. Long-range electrostatic interactions were calculated with the Particle-mesh Ewald method [27] and Lennard-Jones repulsion and dispersion terms with a Cut-Off scheme. All systems were minimized using a combination of Steepest Descent and Conjugate Gradient algorithms until the potential energy reached a value equal to or lower than 239 kcal mol^-1^ nm^-1^.

After a thermalization step, the system was ready to initiate production simulations in an ensemble at constant temperature and pressure (NPT). Temperatures were maintained at 310 K with a time constant of 0.5 ps, and pressure was maintained at 1 bar with a time constant at 0.4 ps. Atomistic simulations were carried out with a time step of 2 fs, while 20 fs was used for the CG simulations.

The stability of Mg^2+^ ions within the five-fold axis was assessed by calculating the PMF of the transition of the ion along the pore. To achieve this goal, we used USM. The ion was positioned at regular intervals of 0.1 nm along the reaction coordinate with a 717 kcal mol^-1^ nm^-1^ force constant. In some windows, the force constant was raised to 2390 kcal mol^-1^ nm^-1^ to prevent ions from leaving the desired position. A total of 40 windows were used. The simulation was performed using a single penton in atomistic detail. The technique g_wham [28] was used to obtain the PMF of the ion from the simulations.

To complete this study, a series of simulations were performed in which Mg^2+^ was positioned along the reaction coordinate to observe its behavior. These sets of calculations were simulated for 2 ns. Only simulations in which the ion remained inside the pore were continued up to 100 ns.

The Gromacs g_densmap subroutine was used to obtain 2D water densities maps. To achieve this goal, RMSD of the water molecule coordinates of each simulation was analyzed to determine the portions of the simulation that were considered stationary and then used to obtain the maps. The pore radius was measured with the program HOLE [29].

### Quantum multilayered simulations

To evaluate proton transport, or hydroxyl generation, through the channel characterized by means of MD simulations, hybrid n-layered integrated different levels of molecular orbital (ONIOM) calculations [30] were performed using the GAUSSIAN 09 (G09) package [31]. These simulations utilized density functional theory (DFT) formalism combined with a semi-empirical (SE) method in two layers (DFT/SE).

The region employed for the QM calculation was a cylindrical zone comprising the amino acids lining the cavity at the five-fold symmetry axis. The inner wall of this region includes 5 rings of five-fold related residues: Val 1166, Thr 1667, Gln 3014, Val 3012 and Thr 3010. A Mg^2+^ and several water molecules were also included in this model, resulting in a total number of 563 atoms for the simulations. For the QM calculations, the initial atomic coordinates were determined from the SPC-solvated pentameric structure obtained after 100 ns of MD simulation. Given that Gromos is a united atom force field, those implicit hydrogens in the MD simulation were added explicitly by means of the program Gaussian View included in the Gaussian package [31]. On the one hand, the DFT region comprised the Mg^2+^ cation, 38 water molecules, four OH^-^ ions and 3 glutamine residues coordinated with the Mg^2+^ ion. Three of the OH^-^ ions were located in the outer bulk solvent at arbitrary positions. The fourth hydroxyl was placed at the position corresponding to a water molecule with a longer residence time per the MD calculations. This last molecule coincided with the outermost water along the water wire formed after the MD simulation. B3LYP hybrid exchange-correlation functional [32–34] and molecular orbitals expanded with the 6-31G** Gaussian basis set were used in this layer. On the other hand, the semi-empirical region (SE) included the remaining amino acids forming the inner wall of the channel. These residues have been described by the AM1 method available in G09 [35,36]. Thus, all atoms involved in the proton channel were modeled using a high-level quantum approach. In contrast, the second layer, which was formed by the nearest neighboring amino acids, was also represented by quantum mechanical calculations (SE region).

Atoms in the DFT region were completely relaxed, while the corresponding ones in the SE region remained static. In these calculations, each molecule was treated separately, either with the DFT or the SE approach, and no special treatment for the DFT/SE boundaries was required. Thus, the DFT/SE boundaries did not include atomic bonds.

To determine the activation energy, the Synchronous Transit-Guided Quasi-Newton (STQN) Method developed by H. B. Schlegel and coworkers [37] was used. For minimizations, it performs optimization by default using redundant internal coordinates. Unlike other methods, STQN does not require a guess for the transition structure; instead, the reactant and product structures are the input. Full vibrational frequency analysis with only one imaginary frequency assures that the obtained geometry corresponds to a saddle point.

The processes for both the energy minimization and TS calculations were repeated until the convergence criteria reached the standard cutoff values, i.e., 0.00045 Hartrees/Bohr for the maximum force component, 0.0003 Hartrees/Bohr for the root-mean square force, 0.0018 Å for the maximum displacement and 0.0012 Å for the root-mean-square displacement.

## Discussion

More than three decades of structural studies on various picorna-like viruses, many of them performed on poliovirus (PV) and rhinovirus (HRV), has resulted in the elucidation of certain aspects of capsid destabilization and RNA release [38–41]. Nevertheless, we are still far from achieving a satisfactory description of the events that trigger and link these two processes in non-enveloped viruses. Several of the questions that remain unanswered are as follows: What are the external and internal factors that destabilize the capsid upon cell attachment or internalization? Does the RNA exit the capsid by being extruded through a small orifice in the capsid, or is it released without unwinding, i.e., in its compact folded form? These questions have been partially addressed in several works using both described picornaviruses as models, and they concluded that the RNA exits the viral capsid through small orifices [38–42]. In accordance with this model of genome externalization, a recent study has shown that the small structural protein VP4 from HRV creates small pores in model lipid membranes [43]. This observation is consistent with the liberation of the RNA by extrusion since the passage of the cell membrane could also occur through the small aperture created by VP4. However, one of the main prerequisites underlying the hypothesis of the RNA extrusion model is the need for the existence of a singular point within the viral capsid. This singular point would anchor the genome in a position in such a way that one of its extremes remains just beneath the capsid hole, thus requiring the icosahedral symmetry of the virion to be broken. This hypothesis remains to be experimentally confirmed.

Our previous studies examining TrV [7,20,44] have shown that the process of genome release is not consistent with the extrusion mechanism observed in PV and HRV [38–41]. This difference was also reported recently in Israeli acute bee paralysis virus [45]. Additionally, experimental data previously reported by our group have shown that an alkaline pH induces weakening of capsid-RNA interactions and that the genome itself can promote capsid opening to be released [7]. Moreover, we also observed that the mechanism of cell permeation by TrV VP4 could differ from that postulated for HRV [43] in the sense that instead of making small perforations, it produces large dynamic pores in model membranes [44]. This study showed that concomitantly with the pH effect on TrV capsid destabilization, the efficiency of VP4 permeation of membranes increased with alkalinization of the medium. In summary, all of our previous observations indicate that the alkaline pH of the solvent triggers key processes in TrV, such as capsid destabilization, genome release, and enhancement of the cell wall disruption.

### The five-fold cavity is a hydrophobic nanopore

Our MD calculations allowed the elucidation of the multiple roles of the solvent in the narrow cavity that traverses the TrV capsid. We observed a hydrophobic gate inside the pore that formed along the capsid five-fold axis, and this empty space prevented solvent exchange between the interior and exterior of the virion. This feature has not been described in viral capsids, but it has been observed and widely studied in membrane ion channels and other non-biological systems [46–49].

The assumption based on crystallographic data that a Mg^2+^ is on the five-fold axis, coordinated to five Gln3014 residues, removes the hydrophobic gate property, allowing for a fully hydrated cavity. Moreover, Mg^2+^ coordinates with incoming water molecules and the oxygen of neighboring amino acids and creates a *water wire* that allows for the connection between the external region and the capsid interior. Hence, MD calculations provide strong support to rationalize the function of the putative ion observed not only on both CrPV and TrV atomic structures but also in other icosahedral viruses. Thus, Mg^2+^ would allow for the outer and the inner capsid regions to become connected through a chain of 8 water molecules that extend approximately 2 nm (Figs 3 and 4). These results suggest the concept of "*hydrophobic gaiting*” for viruses, which would be controlled by the concentration of Mg^2+^, the most abundant intracellular divalent cation. Under this scenario, a first step for virus maturation would be gate “opening” by Mg^2+^ binding, which would be very much dependent on the cell, since it is well-known that different cells maintain different concentrations of Mg^2+^.

### Origin and function of Mg^2+^ at the five-fold axis

It has been noted that mature (full) TrV wild type capsids have open pores with sufficient space to allocate a cation [10], rather than pores that are partially occluded by Gln3014 as observed in empty TrV capsids [20]. The last observation indicates that TrV structural proteins not necessitate cations to assemble nor to maintain the capsid integrity. As a result of the simulations described in this manuscript, we can infer that the putative cation located in this position would play a functional role in hydration and proton conduction through the five-fold channel, and not a structural role as suggested by other authors [50]. Given the observation that the putative Mg^2+^ is trapped between two energy barriers, it is unlikely that this cation enters the specified pore center from either pore entrance, reinforcing the idea that the ion is taken up from the medium during the virus assembly process and released upon genome exit.

### Mechanism of TrV capsid pH sensing

It has been shown in other systems [47,49] that channel hydration is fundamental for favoring the passage of ions by decreasing the energetic barrier for ion translocation. Computational investigations and several experimental approaches have focused on different aspects of water-mediated proton transfer in biological systems. Some examples are the gramicidin A transmembrane channel [51–53], bacteriorhodopsin [54–56], cytochrome C oxidase [57,58], influenza AM2 proton channel [59], green fluorescent protein (GFP) [60], the voltage-gated proton channel HV1 [61], carbonic anhydrase [62] and so-called bioprotonic devices [63].

One of the major QM limitations in modeling biological systems is to represent the solvent complexity at the atomic level; this limitation is observed not only because of the different ions that can be present but also due to the large number of atoms needed to mimic a real physiological pH. Indeed, solely to represent a system at a neutral pH would require a model of more than 3x10^7^ atoms. Since our model is much smaller, our results do not correspond to a real situation, but we can assume that they qualitatively correspond to the pH situation we aimed to describe.

Our QM analysis showed that through a Grotthuss-like mechanism, proton holes could traverse along the water wire (Figs 3 and 4A). This was found to be a concerted transport that moved stepwise proton holes against the pH gradient and protons in the opposite direction. This movement is energetically favored from the outer bulk solvent up to the water molecule coordinated to the Mg^2+^. At this point, a low barrier prevented the continuation of the proton hole towards the internal portion of the water wire (Fig 3E, TS, and Fig 4A). Nevertheless, because this barrier is on the same order as the average kinetic energy of a molecule at room temperature, the hole could continue along its way. From this point, the downward transit of proton holes was energetically favored and reached the internal bulk solvent in two more jumps. In this way, the internal OH^-^ concentration could increase to equal the pH at the capsid exterior.

Additionally, the cation and its three coordinated water molecules formed a barrier that impeded the transit of protons into the internal solvent. *Thus*, *the channel structure acted as a 'diode' since it only permitted the exit but not the entrance of protons into th*e *capsid*.

It can be argued that unidirectional proton passage is not an intrinsic feature of the channel structure, but the result of the simulation conditions mimicking an alkaline or acidic pH. Thus, the likely solvent pH turns the channel into a ‘diode’, allowing proton passage in only one direction. However, it is clear from our calculations that disruption of the water wire is the factor that impedes protons from entering further into the channel. This disruption of connectivity is a consequence of the electrostatic repulsion exerted by Mg^2+^ on the hydronium. Thus, we can conclude that the two factors making the five-fold axis cavity to act as a proton diode are the limited space of the hydrophobic neck and the positive ion trapped in it.

In summary, the leakage of protons through the five-fold cavity is unidirectional and a consequence of the presence of the Mg^2+^. This finding is consistent with the observed sensitivity of the TrV virions to alkaline environments and resistance to acidic pH values [6,7] and constitutes the first mechanistic model to explain the sensitization of viral capsid to alkaline pH values.

### Mechanism of TrV capsid destabilization

The exit of protons through the five-fold cavity permits a plausible mechanism by which the concentration of hydroxyl ions inside the capsid can be increased. Since mature TrV virions are assembled in the cytoplasm of the infected cells, the internal pH condition should be neutral. Once released into the extracellular space, the viral particles can encounter an alkaline environment, after which the capsid proton channels are activated, enabling the internal pH to increase. In one of the possible scenarios, if the pH inside the capsid reaches values of approximately 8.5, which is the pH limit for RNA nucleotides to start to titrate, the hydroxyl ions will compete with the genome for binding the counterions. Under these conditions, RNA is no longer fully neutralized, and the electrostatic repulsion between phosphates induces its unfolding, thus causing the appearance of an internal pressure. In fact, we have previously demonstrated that in virions exposed to an alkaline pH, the RNA is the molecule that promotes capsid destabilization and its disassembly [7]. Although the complete process of genome uncoating most likely occurs in the cell cytoplasm, the extracellular alkaline condition could induce a softening of the viral capsid. This phenomenon has been observed as a prerequisite for effective infection of retroviral particles [64], and for the insect viruses IABPV [45] and Helicoverpa armigera stunt virus (*Alphatetraviridae*; *Omegatetravirus*) [65]. Moreover, the transient opening of the capsid could permit the egress of the internal small protein VP4, the hydrophobic peptide that is thought to be responsible to induce host cell membrane permeabilization in human rhinovirus [43] and in TrV [44].

### Mechanism of TrV capsid dismantling and genome release

The last step of our simulation process was to evaluate the effect produced on the capsid due to repulsive electrostatic forces generated inside the capsid. Our model consisted of the TrV capsid filled with solvent with increasing amounts of anions, which not surprisingly leads to the disruption of the protein shell. After a certain number of anions were added into the interior of the capsid, the simulation showed a rapid increase in the gyration radius (Fig 5B, 5C and 5D). Moreover, this occurred by opening the capsid through the frontier between pentamers, phenomenologically in agreement with a previous energy calculation and experimental data showing that the disassembly of the capsid occurred while maintaining the integrity of the pentons [7]. Interestingly, the crevices observed during the simulations are sufficiently large to allow the simultaneous passage of several solvated Cl^-^. This separation between pentons opened breaches that are large enough to allow the escape of RNA while even maintaining its secondary folding and, perhaps, part of its tertiary structure.

Thus, our calculations support capsid cracking as a process that allows genome egress, which is an alternative mechanism to the previously mentioned extrusion model.

Finally, in our modelization the limit after which the capsid is dismantled is achieved when the internal charge overpasses 200 anions (that corresponds to an internal charge density of 2.8x10^-5^ e^-^/Å^3^). However, we notice that such number of negative charges could be provided by deprotonation of the VP1-4 proteins N-termini, a situation that can occur when the internal pH is alkaline. This charge inversion at the capsid internal face could not only contribute to the capsid destabilization, but also to dwindle the forces that operated during genome encapsidation. To relax these protein-RNA interactions is a condition necessary to detach and then release the genome from the viral capsid proteins.

In summary, here we simulated different steps of the disassembly mechanism of the TrV viral capsid by employing quantum, classical and CG modelization approaches. Our model suggests the presence of hydrophobic (water/proton) gates in viral capsid channels. The presented multiscale computational calculation assigns a functional role to the putative metal ion on the five-fold axis and provides a rationale for the mechanism of capsid pH sensitivity, as well a novel mechanism of unidirectional proton conduction. Moreover, our model links this pH sensitization to the appearance of internal electrostatic forces that would drive the destabilization and disassembly of the TrV viral capsid, which are key steps in permitting genome release.

## Supporting information

S1 Table **Column one.** The N-terminal aminoacids for VP1-3 correspond respectively to Val1001, Lys2009, and Ser3001 [1]. **Column two.** Radial distances from the capsid center to the N-atom of the N-terminal residue corresponding to TrV capsid proteins. **Column three.** Volume of the region comprised between two radial distances. **Column four.** Computed charge density within the spherical shell in the hypothetical case that all N-termini comprised in it where deprotonated. NOTE: The first amino acid of VP2 that is visible into the electron density is Lys2009, indicating that the true radial distribution of the 60 copies of this protein N-terminus is unknown. Nevertheless, due to steric reasons it could be estimated that all of them are at few Ångströms from N-Lys2009.
(DOCX)

S1 Fig OH^-^ ion distributions at the external bulk capsid solvent region. **A.** This pannel shows the distribution of OH^-^s corresponding to Fig 3. **B**. An alternative selection in the position of the three outer OH^-^ shown in **A** gives a similar result for the proton hole migration. **C**. The computed energies for the three proton jumps when an outer single OH^-^ is included are equivalents to those found when three OH^-^ s are present. OH^-^ ions are indicated with red haloes. Numbers correspond to approximate energy values (in kcal/mol) for the proton jump between two adjascent water molecules. The green sphere represents the Mg^+2^ ion.
(DOCX)

S2 Fig N-termini of TrV capsid proteins. **A.** Surface model of two adjascent pentamers shown from the capsid interior. The N-termini of the three major structural proteins VP1-3 face the capsid interior and are exposed to the internal solvent [1]. The smallest protein VP4 is disordered (not shown) and most likely close to the five-fold axis and in close contact with the RNA. **B**. Lateral view of the two pentamers shown in **A** cutted along a plane passing the black arrows (the black region represents the cliping of the electron density). All N-termini of VP1-3 proteins lie in a thin spherical region comprised between **r**_**1**_ and **r**_**2**_ (see S1 Table).
(DOCX)

S3 Fig Water models corresponding to different resolutions. From left to right: atomistic water, WT4 (coarse-grained water molecules) and WLS (supra coarse-grained water molecules). The size of the spheres corresponds to the actual van der Waals radii. The detailed description of the interaction parameters is present in ref [2].
(DOCX)
