# Supplementary material for: Multiscale modelization in a small virus: Mechanism of proton channeling and its role in triggering capsid disassembly
Source: PLoS Comput Biol. 2018 Apr 16;14(4):e1006082. doi: 10.1371/journal.pcbi.1006082 (PMC5919690; doi:10.1371/journal.pcbi.1006082)
Supplement: S1 Table — Column one. The N-terminal aminoacids for VP1-3 correspond respectively to Val1001, Lys2009, and Ser3001 [1]. Column two. Radial distances from the capsid center to the N-atom of the N-terminal residue corresponding to TrV capsid proteins. Column three. Volume of the region comprised between two radial distances. Column four. Computed charge density within the spherical shell in the hypothetical case that all N-termini comprised in it where deprotonated. NOTE: The first amino acid of VP2 that is visible into the electron density is Lys2009, indicating that the true radial distribution of the 60 copies of this protein N-terminus is unknown. Nevertheless, due to steric reasons it could be estimated that all of them are at few Ångströms from N-Lys2009. (DOCX) [file pcbi.1006082.s001.docx]

**Multiscale modelization in a small virus: Mechanism of proton channeling and its role in triggering capsid disassembly**

**SUPPORTING INFORMATION S1 TABLE**

Juan Viso^1,2 π^, Patricia Belelli^1,3 π^, Matías Machado4, Humberto González^4^, Sergio Pantano^4^, María Julia Amundarain^1,2^, Fernando Zamarreño^1,2^,

Maria Marta Branda ^1,3^, Diego M. A. Guérin^5 *^ and Marcelo D. Costabel^1,2 *^

^1^Departamento de Física, Universidad Nacional del Sur (DF-UNS), Avda. Alem 1253. (8000) Bahía Blanca, Argentina

^2^Grupo de Biofísica, IFISUR (UNS/CONICET).

^3^GRUMASICA, IFISUR (UNS/CONICET)

^4^Grupo de Simulaciones Biomoleculares, Institut Pasteur de Montevideo. Mataojo 2020, 11400 Montevideo, Uruguay.

^5^Instituto Biofisika (UPV/EHU, CSIC). Department of Biochemistry and Molecular Biology, University of the Basque Country (EHU). Barrio Sarriena S/N, 48940, Leioa, Vizcaya, Spain

^*^ Corresponding authors: costabel@criba.edu.ar (MDC), diego.guerin@ehu.eus (DMAG: ORCID ID 0000-0001-8504-9636)

^π^ These authors contributed equally to this work.

| Protein | Radial distance (Å) | Volume  (10^4^ Å^3^) | Charge density  (10^-4^ e^-^/Å^3^) |
| --- | --- | --- | --- |
| VP3 | 118.9 (r_1_) | --- | --- |
| VP1 | 117.7 (r_3_) | r_1_ and r_3_: 682 | 2.6 |
| VP2 | 114.8 (r_2_) | r_1_ and r_2_: 693 | 5.7 |

S1 Table. Column one. The N-terminal aminoacids for VP1-3 correspond respectively to Val1001, Lys2009, and Ser3001 [^[[1]](#endnote-1)^]. Column two. Radial distances from the capsid center to the N-atom of the N-terminal residue corresponding to TrV capsid proteins. Column three. Volume of the region comprised between two radial distances. Column four. Computed charge density within the spherical shell in the hypothetical case that all N-termini comprised in it where deprotonated. NOTE: The first amino acid of VP2 that is visible into the electron density is Lys2009, indicating that the true radial distribution of the 60 copies of this protein N-terminus is unknown. Nevertheless, due to steric reasons it could be estimated that all of them are at few Ångströms from N-Lys2009.

1. [] Squires G, Pous J, Agirre J, Rozas-Dennis GS, Costabel MD, Marti GA, et al. Structure of the Triatoma virus capsid. Acta Cryst D Biol Crystallogr. 2013; 69(Pt 6):1026-1037. [↑](#endnote-ref-1)
