# Supplementary material for: Multiscale modelization in a small virus: Mechanism of proton channeling and its role in triggering capsid disassembly
Source: PLoS Comput Biol. 2018 Apr 16;14(4):e1006082. doi: 10.1371/journal.pcbi.1006082 (PMC5919690; doi:10.1371/journal.pcbi.1006082)
Supplement: S1 Fig — A. This pannel shows the distribution of OH-s corresponding to Fig 3. B. An alternative selection in the position of the three outer OH- shown in A gives a similar result for the proton hole migration. C. The computed energies for the three proton jumps when an outer single OH- is included are equivalents to those found when three OH- s are present. OH- ions are indicated with red haloes. Numbers correspond to approximate energy values (in kcal/mol) for the proton jump between two adjascent water molecules. The green sphere represents the Mg+2 ion. (DOCX) [file pcbi.1006082.s002.docx]

**Multiscale modelization in a small virus: Mechanism of proton channeling and its role in triggering capsid disassembly**

**SUPPORTING INFORMATION S1 FIGURE**

Juan Viso^1,2 π^, Patricia Belelli^1,3 π^, Matías Machado4, Humberto González^4^, Sergio Pantano^4^, María Julia Amundarain^1,2^, Fernando Zamarreño^1,2^,

Maria Marta Branda ^1,3^, Diego M. A. Guérin^5 *^ and Marcelo D. Costabel^1,2 *^

^1^Departamento de Física, Universidad Nacional del Sur (DF-UNS), Avda. Alem 1253. (8000) Bahía Blanca, Argentina

^2^Grupo de Biofísica, IFISUR (UNS/CONICET).

^3^GRUMASICA, IFISUR (UNS/CONICET)

^4^Grupo de Simulaciones Biomoleculares, Institut Pasteur de Montevideo. Mataojo 2020, 11400 Montevideo, Uruguay.

^5^Instituto Biofisika (UPV/EHU, CSIC). Department of Biochemistry and Molecular Biology, University of the Basque Country (EHU). Barrio Sarriena S/N, 48940, Leioa, Vizcaya, Spain

^*^ Corresponding authors: costabel@criba.edu.ar (MDC), diego.guerin@ehu.eus (DMAG: ORCID ID 0000-0001-8504-9636)

^π^ These authors contributed equally to this work.

**Proton-hole migration with different solvent OH^-^ distributions**


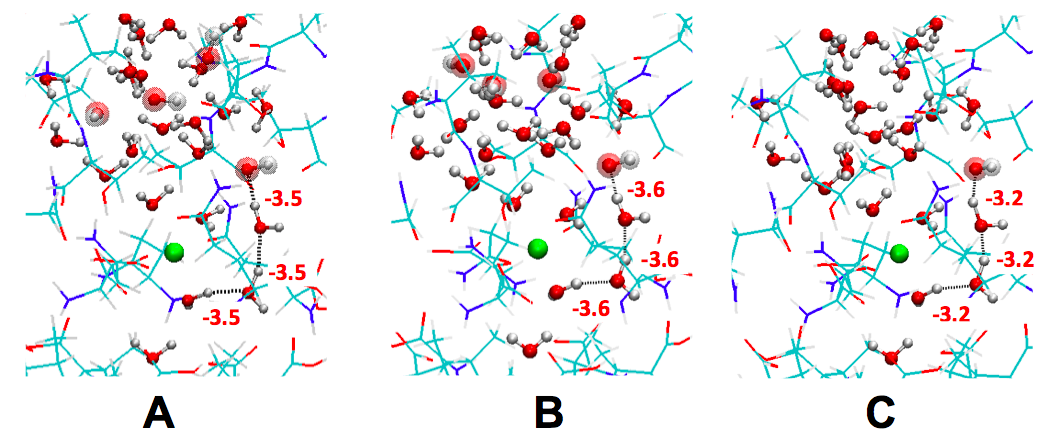


**S1 Figure.** **OH^-^ ion distributions at the external bulk capsid solvent region.** **A.** This pannel shows the distribution of OH^-^s corresponding to Fig. 3. **B**. An alternative selection in the position of the three outer OH^-^ shown in **A** gives a similar result for the proton hole migration. **C**. The computed energies for the three proton jumps when an outer single OH^-^ is included are equivalents to those found when three OH^-^ s are present. OH^-^ ions are indicated with red haloes. Numbers correspond to approximate energy values (in kcal/mol) for the proton jump between two adjascent water molecules. The green sphere represents the Mg^+2^ ion.
