# Supplementary material for: Multiscale modelization in a small virus: Mechanism of proton channeling and its role in triggering capsid disassembly
Source: PLoS Comput Biol. 2018 Apr 16;14(4):e1006082. doi: 10.1371/journal.pcbi.1006082 (PMC5919690; doi:10.1371/journal.pcbi.1006082)
Supplement: S2 Fig — A. Surface model of two adjascent pentamers shown from the capsid interior. The N-termini of the three major structural proteins VP1-3 face the capsid interior and are exposed to the internal solvent [1]. The smallest protein VP4 is disordered (not shown) and most likely close to the five-fold axis and in close contact with the RNA. B. Lateral view of the two pentamers shown in A cutted along a plane passing the black arrows (the black region represents the cliping of the electron density). All N-termini of VP1-3 proteins lie in a thin spherical region comprised between r1 and r2 (see S1 Table). (DOCX) [file pcbi.1006082.s003.docx]

**Multiscale modelization in a small virus: Mechanism of proton channeling and its role in triggering capsid disassembly**

**SUPPORTING INFORMATION S2 FIGURE**

Juan Viso^1,2 π^, Patricia Belelli^1,3 π^, Matías Machado4, Humberto González^4^, Sergio Pantano^4^, María Julia Amundarain^1,2^, Fernando Zamarreño^1,2^,

Maria Marta Branda ^1,3^, Diego M. A. Guérin^5 *^ and Marcelo D. Costabel^1,2 *^

^1^Departamento de Física, Universidad Nacional del Sur (DF-UNS), Avda. Alem 1253. (8000) Bahía Blanca, Argentina

^2^Grupo de Biofísica, IFISUR (UNS/CONICET).

^3^GRUMASICA, IFISUR (UNS/CONICET)

^4^Grupo de Simulaciones Biomoleculares, Institut Pasteur de Montevideo. Mataojo 2020, 11400 Montevideo, Uruguay.

^5^Instituto Biofisika (UPV/EHU, CSIC). Department of Biochemistry and Molecular Biology, University of the Basque Country (EHU). Barrio Sarriena S/N, 48940, Leioa, Vizcaya, Spain

^*^ Corresponding authors: costabel@criba.edu.ar (MDC), diego.guerin@ehu.eus (DMAG: ORCID ID 0000-0001-8504-9636)

^π^ These authors contributed equally to this work.

**Distribution of VP1-3 N-termini**


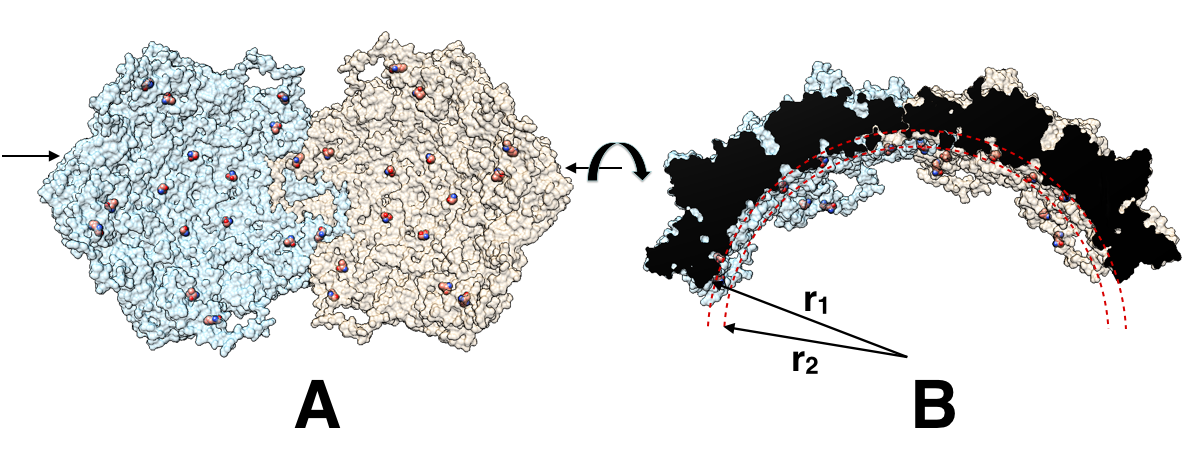


**S2 Figure.** N-termini of TrV capsid proteins. **A.** Surface model of two adjascent pentamers shown from the capsid interior. The N-termini of the three major structural proteins VP1-3 face the capsid interior and are exposed to the internal solvent [^[[1]](#endnote-1)^]. The smallest protein VP4 is disordered (not shown) and most likely close to the five-fold axis and in close contact with the RNA. **B**. Lateral view of the two pentamers shown in **A** cutted along a plane passing the black arrows (the black region represents the cliping of the electron density). All N-termini of VP1-3 proteins lie in a thin spherical region comprised between **r_1_** and **r_2_** (see S1 Table).

1. Squires G, Pous J, Agirre J, Rozas-Dennis GS, Costabel MD, Marti GA, Navaza J, Bressanelli S, Guérin DM, Rey FA (2013) Structure of the Triatoma virus capsid *Acta Cryst D Biol Crystallogr* 69(Pt 6):1026-1037. [↑](#endnote-ref-1)
