# Supplementary material for: Multiscale modelization in a small virus: Mechanism of proton channeling and its role in triggering capsid disassembly
Source: PLoS Comput Biol. 2018 Apr 16;14(4):e1006082. doi: 10.1371/journal.pcbi.1006082 (PMC5919690; doi:10.1371/journal.pcbi.1006082)
Supplement: S3 Fig — From left to right: atomistic water, WT4 (coarse-grained water molecules) and WLS (supra coarse-grained water molecules). The size of the spheres corresponds to the actual van der Waals radii. The detailed description of the interaction parameters is present in ref [2]. (DOCX) [file pcbi.1006082.s004.docx]

**Multiscale modelization in a small virus: Mechanism of proton channeling and its role in triggering capsid disassembly**

**SUPPORTING INFORMATION S3 FIGURE**

Juan Viso^1,2 π^, Patricia Belelli^1,3 π^, Matías Machado4, Humberto González^4^, Sergio Pantano^4^, María Julia Amundarain^1,2^, Fernando Zamarreño^1,2^,

Maria Marta Branda ^1,3^, Diego M. A. Guérin^5 *^ and Marcelo D. Costabel^1,2 *^

^1^Departamento de Física, Universidad Nacional del Sur (DF-UNS), Avda. Alem 1253. (8000) Bahía Blanca, Argentina

^2^Grupo de Biofísica, IFISUR (UNS/CONICET).

^3^GRUMASICA, IFISUR (UNS/CONICET)

^4^Grupo de Simulaciones Biomoleculares, Institut Pasteur de Montevideo. Mataojo 2020, 11400 Montevideo, Uruguay.

^5^Instituto Biofisika (UPV/EHU, CSIC). Department of Biochemistry and Molecular Biology, University of the Basque Country (EHU). Barrio Sarriena S/N, 48940, Leioa, Vizcaya, Spain

^*^ Corresponding authors: costabel@criba.edu.ar (MDC), diego.guerin@ehu.eus (DMAG: ORCID ID 0000-0001-8504-9636)

^π^ These authors contributed equally to this work.

**Multiscale solvation model**

Here we describe the multiscale solvation approach well suited for large macromolecular assemblies, which, like empty viral capsids, require a massive amount of explicit bulk solvent for a proper description [^[[1]](#endnote-1)^]. This approach is combined with the SIRAH force field (South American Initiative for a Rapid and Accurate Hamiltonian) for Coarse-Grained (CG) proteins [^[[2]](#endnote-2)^] to obtain dynamics insight on the TrV capsid.

Briefly, water molecules feature a tetrahedral shape with vertices at Hydrogen atoms and electronic lone pairs. In solution, this results in a central water molecule surrounded by four neighbors, each placing an oxygen atom at the vertices of a tetrahedron [^[[3]](#endnote-3)^]. This transient structuration inspired the WatFour CG model (WT4, for shortness) in which four interconnected beads replicate the structure of a water cluster. This model can be used to represent aqueous solvation at the CG level within the SIRAH force field [3] and/or for hybrid solvation [^[[4]](#endnote-4)^], being transferable to the most commonly used atomistic models [^[[5]](#endnote-5)^]. Hence, WT4 can be simply considered as higher granularity water. This simple observation suggests the possibility to up scale the granularity to represent supra coarse-grained water (Whatelse, or WLS for shortness) that coexist without modifying the essential properties at each level of representation [2]. WLS is composed by four interconnected beads representing 5 WT4 molecules or 55 atomistic water molecules.


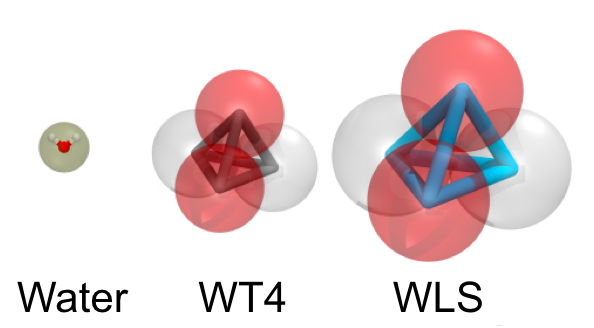


**S3 Figure.** Water models corresponding to different resolutions. From left to right: atomistic water, WT4 (coarse-grained water molecules) and WLS (supra coarse-grained water molecules). The size of the spheres corresponds to the actual van der Waals radii. The detailed description of the interaction parameters is present in ref [2].

1. Machado MR, González HC, Pantano S (2017) MD Simulations of Virus-like Particles with Supra CG Solvation Affordable to Desktop Computers. *J Chem Theory Comput* 13(10):5106–5116. [↑](#endnote-ref-1)
2. Darré L, Machado MR, Brandner AF, Gonzalez C, Ferreira S, Pantano S (2015) SIRAH: A Structurally Unbiased Coarse-Grained Force Field for Proteins with Aqueous Solvation and Long-Range Electrostatics. *J Chem Theory Comput* 11:723-739. [↑](#endnote-ref-2)
3. Darré L, Machado MR, Dans PD, Herrera FE, Pantano S (2010) Another Coarse Grain Model for Aqueous Solvation: WAT FOUR? *J Chem Theory Comput* *6*:3793-3807. [↑](#endnote-ref-3)
4. Darré L, Tek A, Baaden M, Pantano S (2012) Mixing Atomistic and Coarse Grain Solvation Models for MD Simulations: Let WT4 Handle the Bulk *J Chem Theory Comput* 8:3880-3894. [↑](#endnote-ref-4)
5. Gonzalez, H. C.; Darré, L.; Pantano, S. (2013) Transferable Mixing of Atomistic and Coarse-Grained Water Models *J Phys Chem B* 117:14438-14448. [↑](#endnote-ref-5)
